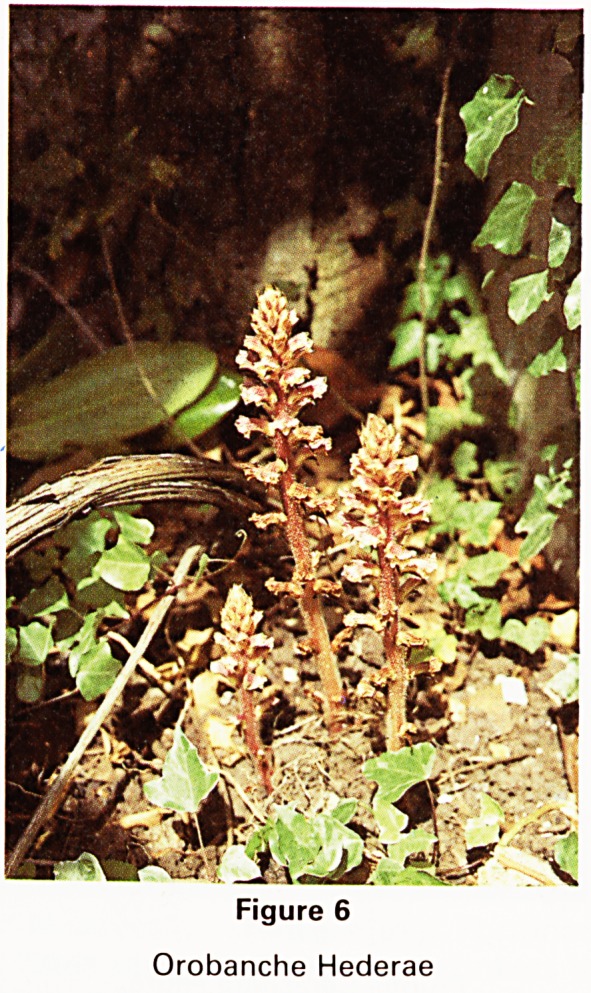# On Gardening

**Published:** 1987-05

**Authors:** T. F. Hewer

**Affiliations:** Emeritus Professor of Pathology, University of Bristol


Bristol Medico-Chirurgical Journal Volume 102 (ii) May 1987

### On Gardening

**T. F. Hewer, M.D., F.R.C.P., F.L.S.**
Emeritus Professor of Pathology, University of Bristol.

When one looks back on a garden that one has planted over a period of forty years it is of some interest to consider what has been worthwhile. There has been time for most things to develop their full capacity and prove their suitability or lack of it. A lot can go wrong. Trees that cast a large area of shade for smaller subjects or come into conflict with others through overcrowding ought to have been given more room or perhaps put to the east side when they should have been put to the west to protect others from the prevailing wind. But if one assumes that proper thought has been given at the very beginning to matters of that sort, and it is not always so, there are still some developments that could hardly have been foretold. Some exceed in beauty or size all that one could imagine, while others of great repute, and perhaps of expense, are bitterly disappointing.

In this note about some of our happier experiences I should like to describe some of our greater joys and successes, especially those that were a surprise. When we came to Henbury in 1946 we found a two acre garden that had suffered years of neglect, with large areas that had not been developed from the wild. Happily there are some things that we spared from our scorched earth campaign and by good fortune we were amply rewarded. First and foremost among these was a very old *Prunus cerasifera* (Fig. 1) the cherry plum or *Myrobalan,* which was fortunately the cultivar *nigra,* not the ubiquitous and inferior *Pissardii.* This tree, close to the low wall at our south boundary, had a trunk with a girth of about four feet and a height of about twenty feet. Its multitude of small white flowers from pink buds in March or earlier, when spared by bullfinches, appear before the leaves. Its girth is now seven feet near the ground and it has spread of forty feet all of which when in flower is magnificent. This tree must have been planted near the middle of last century.

Another very old plant that we inherited was a Winter Sweet, *Chimonanthus praecox (C. fragrans)* growing against a south wall protected from the west wind by a tool shed. In a good summer it sets seeds and we have propagated from it. This plant was introduced into this country by Lord Coventry in 1766. It flowers in midwinter and has a wonderful scent that is so potent that a twig of flowers will scent a room.

There was also an old mulberry, *Morus nigra,* that had survived many vicissitudes losing several large branches but still produces fruit and forms new branches.

In the autumn of 1947, having destroyed almost every other tree and shrub except some fine yews, the tallest of which in the valley is fifty feet high with a lovely trunk, and eliminated all the ground elder by digging and poisoning with sodium chlorate, we were ready to start planting new trees and shrubs. We had surprised our neighbours by leaving the whole area barren for a full year, but it was well worth it.

There are two times of the year when gardens are apt to look dull, unless one does bedding-out or has a herbaceous border—which we do not. One is in midwinter and the other is in July and August when the first flush of flowering cherries and other trees is over. We had therefore not only to plant the early flowering trees and shrubs with spring bulbs below them, but also to provide for those late summer needs. Among the first of these latter that are obtained from Hillier of Winchester was a small rooted branch of the Buck's Eye Tree, as it was originally and oddly called, or *Aesculus parviflora,* from the Southern States of North America. It was introduced to England in 1785 and is one of our greatest treasures. We suspected that this would become quite large and later had proof of this when we saw a fine one in the Botanic Garden in Bath. The photograph of it (Fig 2) in 1974, its thirty fifth anniversary, shows it about half its present size of twenty four feet diameter and ten feet high. It has developed from a large number of slender branches from suckers at ground level. It now needs a little judicious pruning around its periphery to contain it within the limits we had first planned for it. It would show to greater advantage as a single plant in a wide lawn. Apart from being a very beautiful and striking tree it has some great advantages. It flowers luxuriously like a great birthday cake, with hundreds of candles, in August and in November its leaves turn to pure gold. Fortunately it has very small fruits instead of the large conkers that invite damage from the young in the case of other horse chestnuts.

Hydrangeas are other plants that flower well in August and September. There are two groups: the *Hortensias* with globose flowers and the 'Lace caps' with flat ones. We prefer the latter. Some are good in sunny places but most do best in shade or semi-shade and are particularly useful therefore if one is keen on trees. They mostly need careful placing and our most successful in this respect was *Hydrangea Sargentiana* of which we were given three rooted cuttings from my father-in-law, Hiatt Baker's garden at Almondsbury near here in 1951 or thereabouts. These we planted at the bottom of a sheltered valley on the east side of a large yew where they have full sun before noon and semi-shade thereafter. They now form a large group ten to twelve feet high over a spread of twenty five feet. This proved to be a perfect site because it was sheltered from the wind which otherwise breaks off the delicate annual shoots at the end of which the flowers develop. The large flat mauve flowers (Fig. 3) with white peripheral sterile florets are a fine sight in August. Another advantage is that the flowers when they fade become firm brown skeletons that are perfect for indoor decoration and last for years in a dry state.

Hydrangeas produce very fine seed and our *Hydrangea Sargentiana* have never produced seedlings in the ground beneath them but occasionally in walls, either in the mortar or in dry walling, presumably because there is less competition from other plants or slugs to devour them. This hydrangea was introduced from China by seed presented to Kew in 1908 by Professor Sargent of the Arnold Arboretum at Harvard, after whom it was named.

A related species of *Hydrangea, H. aspera,* is a smaller woody shrub that we find less demanding in its site requirements than *H. Sargentiana* and is very beautiful. Its colour varies from pink to light blue with white peripheral florets. The form generally known as *H. villosa* that we have grown between two yews on the main lawn was very large and really magnificent until it was cut down almost to the ground in the hard winter of 1981/2. It has now recovered fairly well with shoots from the reduced old wood and some from the ground.

Another plant from the hydrangea family is *Schizophragma integrifolium.* It also flowers well in August.

**Figure 1**

Prunus Cerasifera Nigra

We have it on the west and north walls of an out-building near the house. It was given to us as a rooted cutting in 1944 from Hiatt Baker's garden. It is much more elegant than *Hydrangea petiolaris* that climbs in the same way. Unfortunately it is difficult to propagate and is rarely seen.

In the winter we have some quite exciting trees and shrubs, chief among them being the *Viburnums.* The ones that perhaps excel all others are a group of hybrids named *Viburnum bodnantense* after the garden in North Wales from which they originally came. Their parents were *Viburnum farreri (V. fragrans) and Viburnum grandiflorum.* They were first produced in 1935 and we have a few different clones which are most attractive and often flower up to the end of November and sporadically throughout the year. They achieve a height of up to ten feet and are spectacular in the sun.

No one who has the space should be without the well known winter flowering cherry, *Prunus subhirtella autumnalis,* which flowers from October onwards. In a mild winter it produces a mass of small white flowers. We have two near each other that merge to produce a fine display. In this December, 1986, they have for us been a background of flowers fifty feet wide and will

**Figure 2**

Aesculus Parviflora probably go on flowering to some extent up to February.

Another group of perennial flowers that we particularly enjoy are the so-called tree-peonies. We have two species that deserve special mention: *Paeonia delavayi and P. lutea,* both originally from the Yunnan province of China. The former may reach six feet in height and has dark red single flowers, while the other is smaller with yellow flowers. Forrest, who collected them, saw some that were natural hybrids of the two species. These have flowers of a great range of colour from dark red (Fig. 4) to pale yellow. They are also pleasantly scented. Hiatt Baker was one of the first to hybridise them in this country in the early 1930s and we have some of his clones which

**Figure 3**

Hydrangea Sargentiana vary greatly in size and colour. They continue to hybridise without any interference from us. They flower in May and June and grow in sun or shade and need little attention.

In other places many species and cultivars of herbaceous peonies, also perennial, are very useful. One of the best is a yellow one with the difficult name of *Paeonia mklosewitschi.* Ours we believe to have hybridised with the white *Paeonia clusii* from Crete which is often called *P. cretica.* This latter was a present from Mr G. P. Baker who collected it in 1933. It is not a strong grower and ours perished, but in its place, which was near *P. mklosewitschi,* there appeared some posthumous seedlings with characters intermediate between the two species.

**Figure 4**

Tree Peonie, Paronia Delavayi

One of our most successful trees, much to be recommended, is *Magnolia kobus.* We bought this as a small single stemmed plant from Hillier in 1946 and put it in front of the Irish Yew facing the house. It grew apace and in March before the leaves it is covered with white star-like flowers similar to those of the much smaller and better known *M. stellata.* It took us by surprise and is now the largest we have ever seen with a girth of 6 feet, becoming four smaller trunks over three feet in girth at five feet from the ground. It is thirty nine feet high with a spread of fifty feet; under it are many snowdrops, so the general effect is very good.

Changing the subject rather abruptly there are some interesting and quite beautiful parasitic plants that are seldom encouraged. In particular there are several species of the *Orobanche* family of which the most beautiful is *Lathraea clandestina* (Fig 5), a bright purple toothwort that grows on the roots of willow and poplar without harming them. It flowers from late March until June and can be propagated by collecting seed from the flowers in June and scattering them at once around other of the same trees. One needs to keep down grass and other weeds as much as possible by hand weeding to create a really spectacular effect.

There are several common broomrapes of the same family, such as *Orobanche hederae* (Fig 6) that grows, as its name implies, on ivy roots. I am hoping to collect some different ones growing on other hosts. None of them has any leaves as they are complete parasites and are restricted to their own host plant.

**Figure 5**

Lathraea Clandestina

**Figure 6**

Orobanche Hederae

One of the great advantages of gardening as a hobby is that it is possible to get the greatest satisfaction out of anything from a window box to a hundred acres. Nowadays it is not easy to maintain a large garden and one discovers additional new pleasure out of the development of parts of it as a wild garden that harbours small birds and butterflies and, in our case, dragonflies that breed in our pond. In any case a garden is a lovesome thing, God wot!*

*See the poem 'My Garden' by T.E. Brown (1830-1897) written when he was a master at Clifton College and lived in Clifton Park.

MY GARDEN

A garden is a lovesome thing, Got wot!
Rose plot,
Fringed pool,
Ferned grot –
The veriest school
Of peace; and yet the fool
Contends that God is not –
Not God! in gardens! when the eve is cool?
Nay, but I have a sign;
'Tis very sure God walks in mine.

Thomas Edward Brown (1830–1897).

## Figures and Tables

**Figure 1 f1:**
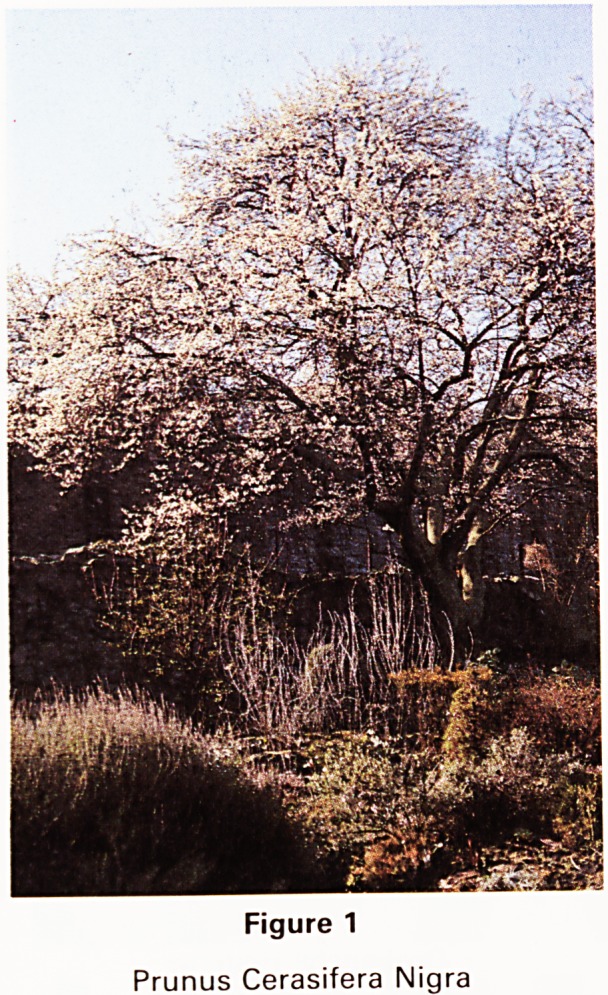


**Figure 2 f2:**
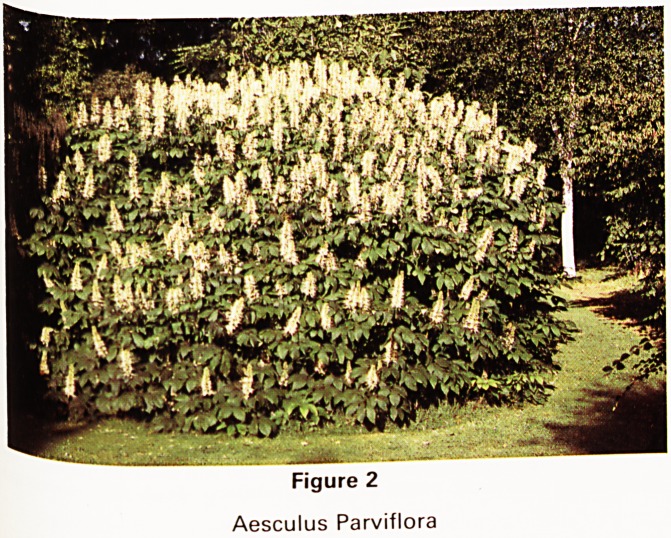


**Figure 3 f3:**
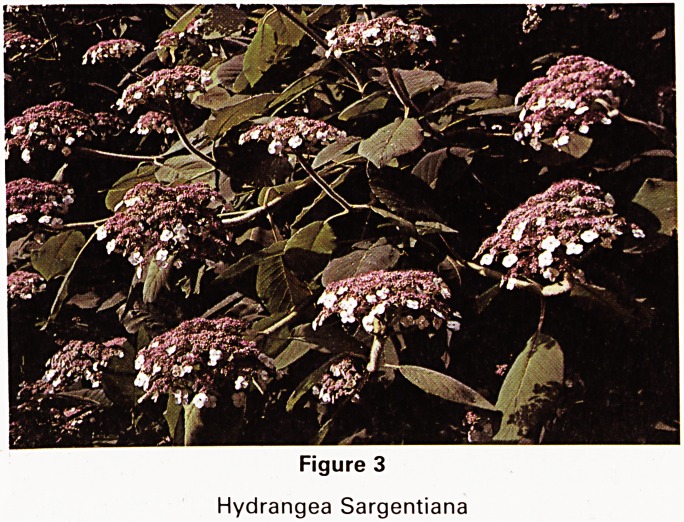


**Figure 4 f4:**
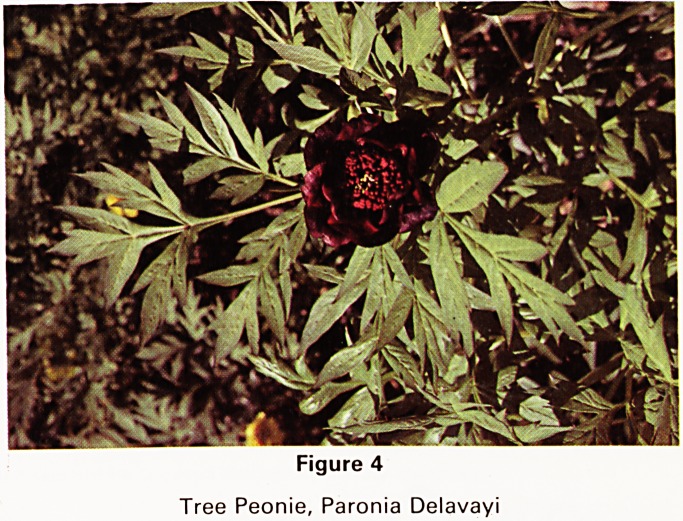


**Figure 5 f5:**
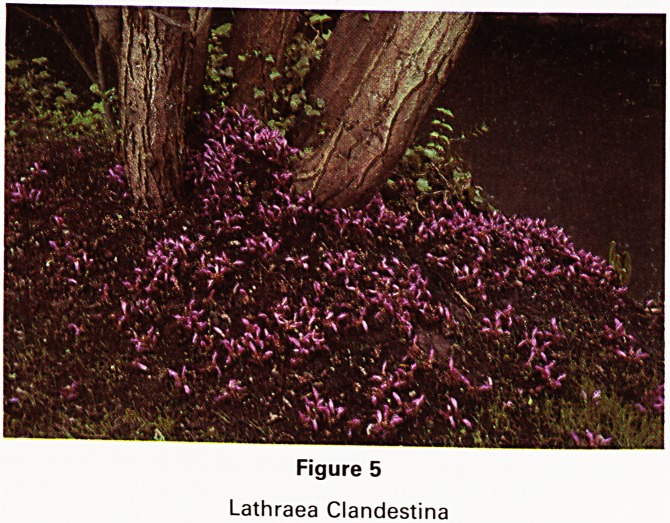


**Figure 6 f6:**